# Neuroinvasion and Inflammation in Viral Central Nervous System Infections

**DOI:** 10.1155/2016/8562805

**Published:** 2016-05-25

**Authors:** Tobias Dahm, Henriette Rudolph, Christian Schwerk, Horst Schroten, Tobias Tenenbaum

**Affiliations:** Pediatric Infectious Diseases, Department of Pediatrics, Medical Faculty Mannheim, Heidelberg University, 68167 Mannheim, Germany

## Abstract

Neurotropic viruses can cause devastating central nervous system (CNS) infections, especially in young children and the elderly. The blood-brain barrier (BBB) and the blood-cerebrospinal fluid barrier (BCSFB) have been described as relevant sites of entry for specific viruses as well as for leukocytes, which are recruited during the proinflammatory response in the course of CNS infection. In this review, we illustrate examples of established brain barrier models, in which the specific reaction patterns of different viral families can be analyzed. Furthermore, we highlight the pathogen specific array of cytokines and chemokines involved in immunological responses in viral CNS infections. We discuss in detail the link between specific cytokines and chemokines and leukocyte migration profiles. The thorough understanding of the complex and interrelated inflammatory mechanisms as well as identifying universal mediators promoting CNS inflammation is essential for the development of new diagnostic and treatment strategies.

## 1. Introduction

Viral central nervous system (CNS) infections can be classified depending on the anatomical site of the inflammation and the entry site of viral pathogens. An infection of the meninges is referred to as meningitis, of the brain as encephalitis, and of the spinal cord as myelitis. When a combination of regions is affected, the terms meningoencephalitis or encephalomyelitis are applied [1]. Viral CNS infections are almost threefold as frequent as bacterial infections with an incidence of 20–30/100.000 per year [2, 3]. Despite an often mild acute phase, fatal outcomes are possible, while the long term impact of viral CNS infections has not been elucidated in detail yet [4].

In healthy individuals various immune cells, which enhance the protection against invading pathogens, are found in small numbers in the CNS. The majority (70–80%) of these immune cells in the cerebrospinal fluid (CSF) are phenotypic CD3^+^ memory T-cells; both CD4^+^ and CD8^+^ subsets are present with around 40–50% and 20–30%, respectively [5]. In contrast, B-cells (dominantly CD27^+^ memory cells), natural killer (NK) cells, dendritic cells (DCs), and perivascular and meningeal mast cells as well as monocytes and polymorphonuclear granulocytes (PMN) are only present in low numbers [6–8]. However, the composition of inflammatory cells in the CNS and especially in the CSF, which can be clinically monitored by lumbar puncture, undergoes constant changes throughout a CNS infection.

An increase in the number of leukocytes within the CSF can especially be noted in patients with viral meningitis, whereas during encephalitis, patients rarely exhibit elevated cell counts in the CSF. During encephalitis, immune cells migrate into the brain parenchyma [9]. The cascade of events following viral CNS infection includes the recruitment of T-lymphocytes, macrophages, and to some extent PMN. An increase in CD4^+^ T-cells, CD8^+^ T-cells, and NK cells has been recorded in the context of viral meningitis. The dominant PMN population seen in early stages of the enteroviral CNS infection, for example, may pave the way for monocytes and lymphocytes [10].

In this review we highlight the role and probable causes of leukocyte migration via the BBB and the BCSFB into the CNS following viral infection. In this context we further describe the associated mediators and their influence on the migration patterns.

## 2. Involvement of the Brain Barriers in CNS Infection

**Figure 1:**
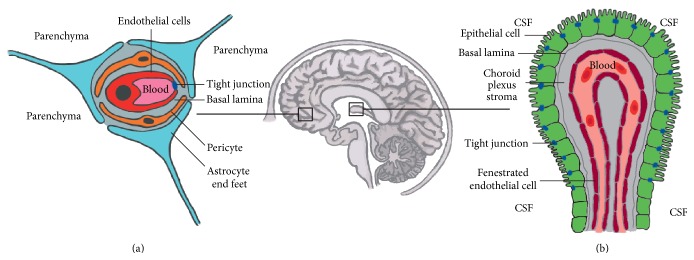
Barriers of the brain. (a) The BBB found between the lumen of the cerebral blood vessels and the brain parenchyma is composed of capillary endothelial cells, astrocytes, and pericytes and obtains its characteristic physical barrier function via linking the endothelial cells through luminal tight junctions. (b) The BCSFB located between the CSF and the fenestrated blood vessels of the choroid plexus. This selective barrier contains polar choroid plexus epithelial cells joined to one another through tight junctions found on the apical side.

To cause CNS infections pathogens must first successfully cross the protective barriers of the brain. The CNS is an immune-specialized site, which maintains a homogenous environment by regulation of entrance and exit of substances through the major brain barriers: the blood-brain barrier (BBB) and the blood-cerebrospinal fluid barrier (BCSFB) [11]. The BBB and BCSFB form metabolic (enzymatic activity on passing molecules), physical (minimizing the paracellular flux), and transport (controlling molecular flux through specialized membrane transporters) barriers [12, 13] (Figure 1).

**Figure 2:**
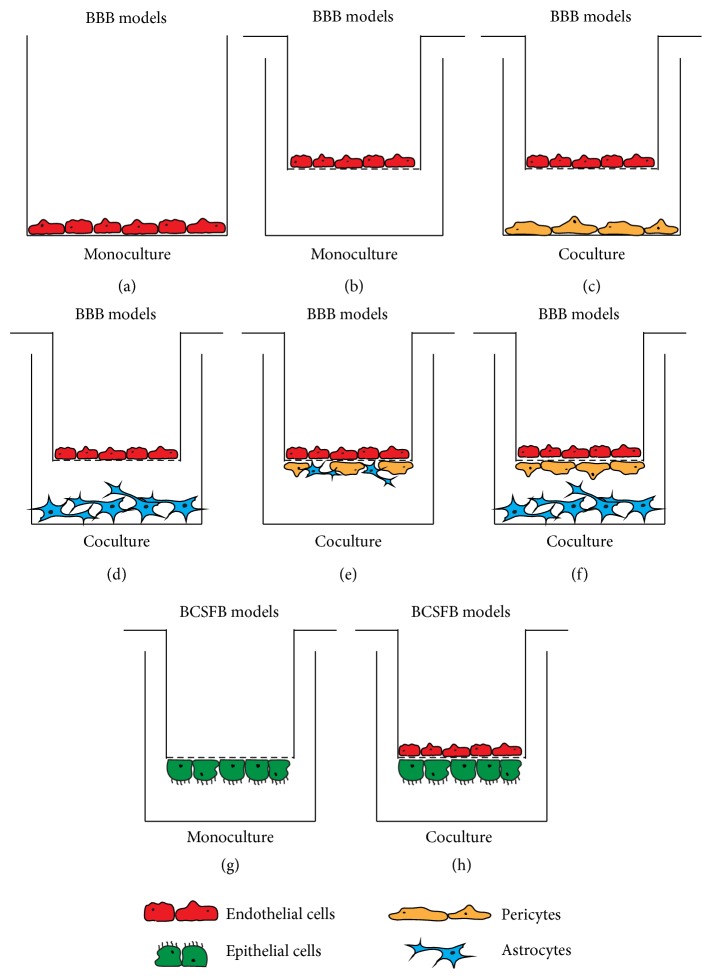
Schematic representation of various* in vitro* BBB and BCSFB models. The BBB models are built up around endothelial cells, whereas the BCSFB models rely on epithelial cell culture. The setup can be a monoculture: endothelial or epithelial cells only (a, b, and g); coculture: endothelial cells with epithelial cells (h) or endothelial cells with pericytes or astrocytes (c and d); and triple-culture: endothelial in combination with astrocytes and pericytes (e and f). These systems can be set up in a “contact” (e, f, and h) or “noncontact” (c and d) manner (“contact”: different cell types have physical contact with each other; “noncontact”: different cell types were not able to physically interact with each other). The monoculture models represented in (a) and (b) can also be used with epithelial cells instead of endothelial cells. The addition of chemical mediators (cytokines and/or chemokines) and virus or immune cells (T-, B-cells, and PMNs, etc.) is possible in both the filter insert and/or well.

The BBB, which has the largest surface area available for communication between the brain and the blood, creates a barrier through a functional unit consisting of endothelial cells, which are joined and stabilized through tight junctions (TJs), astrocyte end feet, and pericytes. This highly selective barrier between the peripheral blood and the brain parenchyma of the CNS consists of specialized cells which in cooperation create a homeostatic environment for cells of the CNS [14, 15] (Figure 2).

The BCSFB is found in the choroid plexus (CP) located in the ventricles of the brain. The epithelium of the CP is mainly responsible for the barrier function of the BCSFB. Apart from maintaining the physical barrier of the CP, the BCSFB, with its array of specific transport channels and a low pinocytotic activity, is involved in para- and transcellular transport of molecules and immune cells between the brain and the cerebrospinal fluid [16]. The endothelial cells within the choroid plexus parenchyma are fenestrated and hardly limit flux of molecules across the endothelial cells [17]. This fenestration is important for the main function of the CP, which is the synthesis of CSF [18].

The paracellular permeability of the BBB and the BCSFB is mainly regulated by two junctional complexes: TJs and adherens junctions (AJs) [19]. The TJs are built and reinforced through a number of junctional proteins such as occludin, claudins, coxsackie adenovirus receptor (CAR), and also junctional adhesion molecules (JAMs), whereas the AJs largely consist of transmembrane cadherins closely linked by alpha, beta, and gamma catenin to the cytoskeleton [19–22]. The primary (direct) or secondary (via host cell signaling events) disruption of those junctional molecules by CNS pathogens can facilitate crossing of virus particles, leukocytes, and inflammatory mediators into the CNS [15].

## 3. Pathogen Entry into the CNS

**Figure 3:**
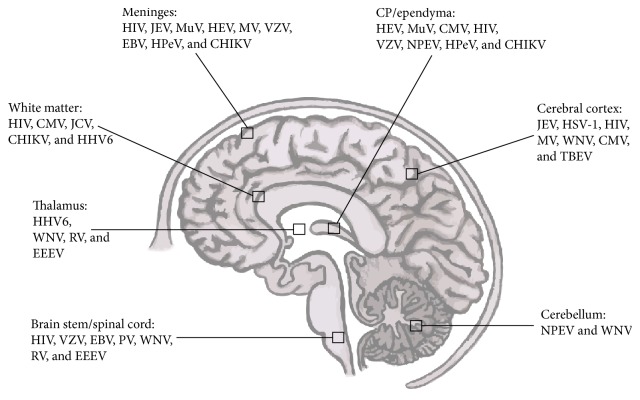
Relevant encephalitis and meningitis causing viruses in humans allocated to the brain region they may affect. CHIKV, chikungunya virus; CMV, cytomegalovirus; EBV, Epstein Barr virus; EEEV, eastern equine encephalitis virus; HHV-6, human herpes virus-6; HIV, human immunodeficiency virus; HPeV, human parechovirus; HSV-1, herpes simplex virus-1; JCV, John Cunningham virus; JEV, Japanese encephalitis virus; MV, measles virus; MuV, mumps virus; NPEV, nonpolio enterovirus; PV, poliovirus; RV, rabies virus; TBEV, tick-borne encephalitis virus; WNV, West Nile virus; VZV, varicella zoster virus.

The brain capillaries and the CP are relevant sites of entry for pathogens such as bacteria, viruses, fungi, and parasites into the CNS. For entry the pathogens must first cross the respective barriers, which are the BBB and the BSCFB. The BBB has been described as an entrance site for bacterial pathogens such as* Neisseria meningitidis (N. meningitidis)* [23, 24] and* Streptococcus pneumoniae (S. pneumoniae)* [25, 26], for fungi such as* Candida albicans (C. albicans)* [27, 28], and also for viruses like West Nile virus (WNV) [29, 30], human immunodeficiency virus (HIV) [31, 32], or herpes simplex virus (HSV) [15, 33]. In contrast, the BCSFB has not received the same amount of attention, due to the lack of adequate* in vivo* and* in vitro* models for this barrier. To date, a range of bacteria as well as viruses were shown to use the BCSFB as an entry site into the CNS, which include* Streptococcus suis (S. suis)* [34–36],* N. meningitidis* [37, 38],* Haemophilus influenzae (H. influenzae)* [39–41], and* Listeria monocytogenes* [42–44] as well as coxsackievirus B3 (CVB3) [45, 46], chikungunya virus (CHIKV) [47], and Echovirus 30 (EV30) [48]. The role of the choroid plexus during infectious diseases has previous been reviewed [49]. However, some viruses are better adapted to invade the CNS through peripheral nerves (poliovirus (PV)) [50–52] or olfactory sensory neurons (herpes simplex virus-1 (HSV-1)) [53, 54] (Figure 3). Limitation to one of these pathways is not obligatory as can be seen with WNV [55] and PV [56]. The most common pathogens causing viral CNS infection are displayed in Table 1.

Due to the diverse structural characteristics of the brain barriers, pathogens developed a range of different strategies to gain access into the CNS via the host cells. Infectious agents were shown to use the transcellular, paracellular, and the “Trojan horse” approaches [75]. Pathogens migrating transcellularly, such as HSV-1, HIV, and EV30, invade directly into host cells to overcome the barrier [48, 76, 77]. For the paracellular transmigration, suggested for HIV-1 [78] and mouse hepatitis virus [79], the pathogens migrate between the TJ molecules. Invasion of pathogens into the CNS whilst internalized in phagocytic host cells has been described as the “Trojan horse” strategy and has been implied to be exploited by many viruses including HIV, CVB3, and WNV [80–83]. However, each pathogen may not use only a single route but rather a combination of strategies [84]. Disruption of the barrier may also lead to increased neuroinvasion and depends on direct cytotoxic effects of the pathogen as well as secondary inflammatory mediators such as cytokines and chemokines, matrix metalloproteinases (MMPs), free radicals, and lipid mediators [15, 85].

Successful invasion of pathogens into the CNS via the brain barriers is usually followed by a strong inflammatory host response resulting in a substantial migration of immune cells to the site of infection to control or eradicate the invading pathogens. Host cells at the brain barriers detect pathogen-associated molecular patterns (PAMPs) in the circulating blood, which leads to activation of immune cells found in close proximity [86]. The BCSFB is believed to be the primary site for immune surveillance of the CNS as it acts as a selective gate, whereas the BBB is designed to function as a strong immunological barrier which hinders leukocyte migration into the parenchyma [87].

## 4. Models to Study Viral CNS Infection

Several* in vitro* models, both static and under flow conditions, as well as* in vivo* models, mainly murine, exist to study the pathogenesis of viral CNS infection. Application of* in vitro* models can facilitate easier handling and may increase the spectrum of potential investigations in comparison to a complex experimental* in vivo* setup. However, in* in vitro *setup, it is barely possible to mimic the extremely complex and interrelated structures of the CNS.* In vitro* models of the BBB can be grouped into two major set ups: (1) single culture models with brain microvascular endothelial cells (BMEC; primary or immortalized cells) and (2) coculture models with, for example, BMEC, astrocytes, and pericytes and/or glia cells [88, 89] (Figure 2).

A commonly used single culture BBB model to study CNS infection is based on human brain microvascular endothelial cells (HBMEC) [89]. For example, Coyne et al. showed a dynamin and caveolin dependent internalization of PV into HBMEC [90]. Conversely, when repeating similar experiments with non-CNS specific cell lines, such as SH-SY5Y or adherent HeLa (S3), the process showed caveolin independence but a tyrosine kinase dependency [91]. This indicates an organ specific mode of entrance into the CNS. A coculture model with HBMEC in combination with human fetal astrocytes was used to investigate HIV-associated encephalitis [92]. The model showed physiological important characteristics such as a functional monolayer with TJs (resulting in a high electrical resistance) and glial fibrillary acid protein (GFAP) reactivity of the endothelial cells and the astrocytes, respectively [92] (Figure 2).

Viral infiltration and subsequent inflammation of the CNS can also have an effect on the functionality of the BCSFB. Several functional* in vitro* models of the BCSFB have been developed including primary and immortalized murine cell lines such as Z310 [93, 94], primary and immortalized porcine choroid plexus epithelial cells (PCPEC), and PCP-R [36, 95, 96] as well as the recently characterized immortalized human choroid plexus papilloma cell line (HIBCPP) [34, 48, 97]. The suitability and limitations of immortalized murine and human cell lines (low transepithelial electrical resistance (TEER) development and uncontentious TJ connections) have been studied intensely and restrictions for broader usage have been encountered [18, 98]. In contrast, the immortalized polar cell line HIBCPP can be used as the first functional human* in vitro* model of the BCSFB [34, 95, 99]. Moreover, through the establishment of an inverted cell culture system with polar cells (Figure 2), it is possible to infect the cells from the physiologically relevant basolateral side (blood) and to investigate viral infection leading to migration of leukocytes from the basolateral (blood) to the apical side (CSF). This can be brought even closer to* in vivo* conditions through addition of cytokines and chemokines. Schneider et al. [48], for example, used the HIBCPP model in combination with physiologically relevant chemokines such as CXCL3 and CXCL12 to investigate the complex interactions and cooperation of T-lymphocytes after infection of HIBCPP with EV30.

Despite a range of* in vitro* and* in vivo* models, there are still many open questions about the pathogenesis of viral CNS disease. The exact role of the CNS barriers and specific leukocytes is yet to be validated. Strain specificity and different infectability of the cell in the CNS complicate the development of adequate infections models. Moreover, for many viruses we lack* in vivo* proof of specific host cell infection in animal models, which minimizes the validity of the deductions adaptable to human infection. A comparative analysis between the data obtained from animal experiments and human data remains difficult [100–102].

## 5. Cytokines and Chemokine Involvement in Viral CNS Infection

Cytokines are involved in almost all biological processes and maintain an important regulatory element. Most cytokines are polypeptide messenger molecules (8–140 kDa), and some may also be glycosylated. Communication and interactions of cells are influenced or controlled through release of cytokines, by the cells themselves or other cells of the organism, that vary according to different stimuli [103]. They are involved in both proinflammatory and anti-inflammatory processes, referred to as T-helper 1- (Th1-) and T-helper 2- (Th2-) cytokines, respectively. Their biological function in the context of inflammatory response can be differentiated into four major groups: (1) innate immunity (IL-1, IL-5, IL-6, and IL-8); (2) management of inflammatory processes (IL-1, IL-4, and TGF-*β*); (3) lymphocyte activation and proliferation (IL-2 and IL-4); and (4) leukocyte growth mediation (IL-1, IL-3, IL-5, and IL-6). If cytokines are involved in chemical attraction of cells they are referred to as chemokines, which are small molecules (~8–14 kDa) that can further be split into four major subgroups (CXC, CC, XC, and CX3C) depending on their structural organization of conserved cysteine residues in the amino terminus [103–105]. Immune responses, in particular trafficking and development of immune cells, are controlled through the superfluous synthesis and release of cytokines and chemokines [105, 106]. A distinct profile of cytokines and chemokines leads to an effective and specific host defense and promotes leukocyte migration during viral CNS infection. During meningitis and encephalitis, an array of cytokines and chemokines has been demonstrated to be regulated including CCL2, CXCL10, CXCL12, IL-1*β*, and TNF-*α* [107–110].

Interestingly, an upregulation of IL-6 and TNF-*α* cytokines so far primarily associated with bacterial meningitis, has been detected in the cerebrospinal fluid of patients with aseptic viral meningitis. However, IL-6 is also involved in T- and B-cell recruitment [111]. The importance of IL-6 during the life cycle of immune cells has been described for CD4^+^ T-cells. During the maturation process of B-cells, to produce functional antibodies, important steps are stimulated through this proinflammatory cytokine [112].

A crucial chemokine in the context of viral CNS infection is CXCL12, which is the second most highly conserved chemokine ligand and occupies a fundamental role in the immune surveillance of the CNS. Together with its receptors CXCR4 and CXCR7 it encourages leukocyte communication and connection in the perivascular space [113]. However, if a state of disease alters the homeostatic distribution of CXCL12 it can lead to a loss of polar expression of the chemokine, subsequently leading to an accumulation of CXCR4^+^ cells in the brain parenchyma, which may increase the severity of the disease. This has been observed in multiple sclerosis (MS) and its adapted mouse model for experimental autoimmune encephalomyelitis (EAE), where the loss of CXCL12-CXCR4 interaction leads to augmented expression of Th1 inflammatory mediators and amplified parenchymal immune intrusion [114]. CXCL12 was also investigated by Schneider et al. [48] as a chemoattractant of T-lymphocytes. In a human* in vitro* model with HIBCPP cells CXCL12 stimulation led to an increased T-lymphocyte basolateral to apical migration, whereas stimulation with CXCL3 did not induce higher migration rates. A time dependent upregulation of CXCL1, CXCL2, CXCL3, IL-8, and CCL5 after infection with EV30 was observed. Taken together, this indicates an involvement of various other factors in the process of T-lymphocyte transmigration into the infected human CNS.

## 6. Inflammatory Reaction Caused by Specific Viral Pathogens during CNS Infection

In the next paragraphs we describe in more detail the specific inflammatory reactions caused by several viral pathogens during CNS infection known to date. Examples of the Retroviridae, Herpesviridae, Picornaviridae, Flaviviridae, Paramyxoviridae, and Togaviridae families will be discussed in relation to their effects on the CNS immune response and release of mediators.

### 6.1. Retroviridae

Of the family Retroviridae, two main viruses, the human T-lymphotropic virus-1 (HTLV-1) and HIV, are important CNS pathogens, of which we will focus on the latter. Many HIV-1 variants exist, which display a large range of genetic alterations [115]. These can cause numerous neurological disorders, some of which can lead to cognitive, motor, or behavioral disorders, which together are known as HIV-associated neurocognitive disorders (HAND) [116].

HIV has the ability to disrupt the BBB and invade the CNS in the course of primary infection. The mechanisms crucial for HIV-1 infection of the CNS through BBB disruption have been studied extensively in* in vitro* and in* in vivo* models [31, 57, 117]. The virus is able to survive in leukocytes, thereby evading the immune system or antiviral treatment. HIV can “hide” inside T-cells [118, 119] but also inside monocytes, macrophages [120], and dendritic cells [121]. The migration and survival of HIV within leukocytes (“Trojan horse”) is of major advantage for the virus [31, 122]. These infected leukocytes may migrate to areas which cannot be reached with antiretroviral drugs and consequently HIV can persist and further replicate [123].

The composition of the specific monocyte populations can also change during HIV infection. The population of CD14^+^CD16^+^ monocytes (5–10%) found in healthy individuals increases up to 40% following HIV infection. Furthermore, a stronger migration of infected CD14^+^CD16^+^ monocytes, compared to noninfected monocytes, was observed in response to CCL2 in an* in vitro* BBB model consisting of a coculture of HBME and astrocytes [124]. Infected mature monocytes from the vascular system were shown to move into the CNS and release virulence factors, which activate adjacent macrophages, astrocytes, and microglia, which consequently synthesize and release inflammatory cytokines such as TNF-*α*, IL-1*β*, and IL-6. This array of inflammatory cytokines contributes to BBB disruption and to the development of several disorders related to HAND [125]. Experimental data indicate that HIV persists in the tissue of the host organism due to the continuous migration of monocytes from the peripheral vascular system to the inflamed CNS. The monocyte chemoattractant chemokine ligand 2 (CCL2), which binds to CCR2 (on monocytes), has been shown to be strongly increased in the CSF during HIV infection of the CNS which can lead to the migration of both infected and noninfected cells into the CSF [126].

Specific chemokines can also contribute to successful neuroinvasion of the invading pathogen. In healthy individuals the basal levels of CCL2 in the CNS are low. However, HIV-infected monocytes exhibit enhanced CCR2 expression and were demonstrated to migrate and disseminate into the CNS. Furthermore, the data indicate that the virus is involved in assuring constant high expression of CCR2 on peripheral blood mononuclear cells [122]. In the mouse brain it was possible to measure elevated levels of CCL2, CCL3, CCL4, CCL5, and CXCL10 after retroviral infection [127]. In patients with HAND elevated levels of IL-8, CCL2, CXCL10, and G-CSF have been measured in the cerebrospinal fluid (CSF) [128].

Taken together, CCR2-/CCL2-mediated migration of CD14^+^CD16^+^ monocytes into the CNS may be critical during HIV-mediated CNS pathology.

### 6.2. Herpesviridae

Herpesviridae such as herpes simplex virus (HSV), varicella zoster virus (VZV), Epstein Barr virus (EBV), cytomegalovirus (CMV), and human herpes virus-6 (HHV-6) are causes of acute encephalitis and to a lesser extent meningitis [129]. Particularly HSV-1, on which we will focus in the following, is known to be the most common causal agent of sporadic herpes simplex encephalitis (HSE) through infection of neurons in the trigeminal ganglia. HSV infection of dorsal root sensory ganglia may cause lifelong latency [130].

Within an incidence of about 1/250,000 cases, HSE mainly affects small children (6 months to 3 years) and adults older than 50 years [131]. HSE is associated with a mortality rate of up to 50–70% when left untreated, and even with appropriate treatment the disease frequently causes long term-sequelae [132]. Even though HSE has been studied thoroughly, its exact pathogenesis is still only vaguely understood [131].

The process of immune cell migration from the perivascular space to the CNS is directed and amplified through varying expression levels of specific ligands and receptors. The most important receptors during HSV-1 infection are CCR2, CCR5, and CXCR3, which are linked to Th1 polarized activated T- and NK cell recruitment [133, 134]. In a model of experimental autoimmune encephalitis (EAE) and HSV-1 infection, the interaction of CCL5/CCR5 is involved in T-cell recruitment into the CNS. The expression levels of the ligand CCL5, which is presented by T-cells and macrophages, played a crucial role during leukocyte migration [135]. In CCR5^−/−^ mice a significant elevation in the chemokines CCL2, CCL5, CXCL9, and CXCL10 in the trigeminal ganglion and brainstem after HSV-1 infections was observed. Moreover, the increase of the chemokine expression was associated with a significant increase in virus burden and an increase in the infiltration of CD4^+^ and CD8^+^ into the trigeminal ganglion and brainstem [136].

It is know that microglia and blood-borne immune cells such as macrophages, dendritic cells, and T-cells have surveillance functions in the CNS to control the entry of viruses such as John Cunningham virus (JCV), HIV, WNV, and HSV-1 [137]. For example, mice lacking the principle subunit of the type 1 IFN receptor IFNAR-A1 have been shown to have a drastic reduction of viral surveillance, which can be led back to a decline in virus specific cytotoxic T-cells. In contrast, an increase in macrophages within the CNS of IFNAR-A1 deficient mice has been observed [138].

Additionally, the control of migration of immune cells from the blood to the infected tissue area and the maintenance of the unique and homeostatic immunological environment of the CNS can be largely attributed to CX3C chemokine receptor 1 (CX3CR1) and its ligand CX3CL1. The importance of these tightly regulated interactions has been identified in mice deficient in CCR2 and CX3CR1 receptors [131, 139].

In the same line with the abovementioned findings, HSV-1 mediated sequelae were found to be increased in CXCR3-deficient mice [140]. The Th1-mediated immunological response to infection arises from increased expression of CXCR3 on NK, CD4^+^, and CD8^+^ cells. Furthermore, the interaction of CXCR3 with CXCL9 and CXCL10 is important throughout the immunological response. In particular, CXCL10 is strongly expressed in the CNS during early stages of HSV-1 infection, and deficiency has been shown to lead to increased mortality partially attributed to the hindered NK cells recruitment [105, 133]. Finally, clinical studies demonstrated increased levels of CCL2, CCL3, CCL5, and CXCL8 in the CSF of HSV-1 infected humans [141].

In conclusion, particularly CCR2, CCR5, and CX3CR1 have suggested to be relevant during the pathogenesis of HSV CNS disease. Their expression levels, in combination with the availability of their respective ligands CCL2 and CCL5, have been linked to recruitment of activated immune cells at the site of infection in the CNS.

### 6.3. Picornaviridae

The Picornaviridae family contains linear, very small single-stranded RNA viruses, which modify and then utilize intracellular membranes in the host cells to replicate their genomic RNA [142]. After the broad implementation of polio vaccines, nonpolio enteroviruses (NPEV) are the main cause of viral CNS infections worldwide [3, 143]. NPEV are widespread and cause a range of diseases in humans such as meningitis, encephalitis, or meningoencephalitis. Furthermore, high levels of morbidity and mortality in humans can be observed in young children and infants infected with NPEV such as echovirus, enterovirus 71, or coxsackievirus [144].

CVB3, especially, has been used in several studies analyzing the pathogenesis of NPEV CNS infections. The chemokine profile and leukocyte migration have been studied by Tabor-Godwin et al., who showed a crucial involvement of CCL12 (commonly known as a monocyte attractant) during CVB3-infected nestin^+^ myeloid cell migration across the BCSFB into the CNS [80]. They also found that CVB3 is able to infect neural stem cells and maintain viral persistence [46, 145]. These data indicate a “Trojan horse” entrance pathway of CVB3 exploiting its ability to infect nestin^+^ myeloid cells. Additionally, it has been suggested that B-cells may be involved in the spread of the NPEV through the “Trojan horse” mechanism as well [45].

A close interaction between an immunological response and cytokines and chemokines can also be seen during the clinical course of an infection with Picornaviridae viruses. This has been demonstrated through a changing inflammatory profile after EV30 infection. A shift from proinflammatory mediators such as IL-6, IL-8, and IFN-*γ* to the production of IL-10 or TGF-*β*1, known to be anti-inflammatory cytokines, has been demonstrated in the CSF of children [146]. Furthermore, raised levels of IL-8 and CXCL10 in the blood and CSF, respectively, have been measured in patients suffering from brain encephalitis caused by EV71 [147]. The Th1 T-lymphocyte-mediated immunological response to viruses during CNS infection, especially the chemical attraction of Th1 T-cells, has been demonstrated to rely on CXCL10. The prompt expression and release of this mediator allows a controlled initiation of the host response [109]. Additional chemokines were shown to be upregulated in the CSF of Picornaviridae-infected mice such as CCL2, CCL4, CCL5, CCL6, CXCL10, IL-10, and TGF-*β*1 [146, 148]. Their role in human disease has to be further clarified.

Due to the sparse information on cytokine and chemokine involvement during NPEV CNS infection, further studies should be conducted in both* in vitro* and in animal models also involving various clinical isolated and outbreak strains to broaden the understanding of Picornaviridae infections in humans.

### 6.4. Flaviviridae

Flaviviridae viruses are positive sense, single stranded RNA viruses often infecting humans and mammals. Currently, over 70 species are part of this dominantly arthropod-borne, viral family, out of which especially dengue virus, Japanese encephalitis virus (JEV), and WNV cause severe CNS infections in humans [149]. Due to the increasing number of outbreaks caused by WNV over the last years in Europe and North America, we have focused on this species in this review.

The pathogenesis of WNV CNS infections has been studied in a range of animal models including monkey [150], rat [151], hamster [152], and horse [153]. The large array of experimental studies conducted in rodent models and their validity has been reviewed previously [154]. However, so far, neither an ideal animal nor an* in vitro* WNV infection model to study the CNS pathogenesis exists and all have their specific limitations, which will be discussed in this section.

To cause CNS infections the virus must penetrate both neural and extraneural barriers such as the BBB [84]. Interestingly, in an* in vitro* study with primary human BMEC infected with a neurovirulent WNV strain, it has been demonstrated that infection with WNV does not need BBB disruption to facilitate leucocyte transmigration. However, a high expression of cell adhesion molecules correlated with an increased WNV-infected immune cell migration [83].

Successful invasion of WNV into the CNS causes an activation and migration of leukocytes, mainly T-cells and monocytes. Overall, increased T-cell migration during WNV encephalitis has been attributed to the release of IL-1*β*, CXCL10, CCL2, and CCL5 in the brain [113, 155, 156]. Interestingly, varying levels of CXCL10 synthesis throughout the infected CNS can lead to unbalanced inflammatory cell migration. In mice, strain specific WNV infection of cerebellar granule cell neurons but not uninfected neurons led to CXCL10 synthesis. The unbalanced chemoattraction leads to a more dominant immune cell migration to the cerebellum, leaving other parts of the infected CNS less affected [104, 157]. Furthermore, following WNV infection, neuronal secretion of CXCL10 has been shown to recruit CXCR3^+^ CD8^+^ cells [155, 157, 158]. During WNV infection in mice, a more elevated CD8^+^ migration compared to CD4^+^ T-lymphocytes into the CNS could also be observed [158]. A further study by Shirato et al. [159] on chemokine involvement in WNV CNS disease showed a highly elevated chemokine expression profile of CCL3, CCL4, and CCL5 in the CNS of mice infected with a lethal WNV strain isolated in New York. In the infected mouse brain, a vastly enhanced expression of CCL3, CCL4, CCL5, and CXCL10 was observed on mRNA level. This model also allowed demonstration of elevated B-cell and monocyte-activating chemokine (BMAC) levels (known as CXCL14 in humans) during stages of advanced infection.

Recently, the role of CXCL12 has also been addressed in murine WNV models. Due to elevated CXCL12 levels in the perivascular space, leukocytes accumulated but did not penetrate into the CNS directly. Only once was CXCL12 downregulated and subsequently, its concentration was decreased in the perivascular space; immune cells migrated across the brain barriers into the CNS. A functional cooperation between the ligand CXCL12 in the perivascular space and its receptor CXCR4 on the host cells is essential for leukocyte migration into the CNS. This has been underlined through inhibition studies on CXCR4, which overall increased T-lymphocyte entry into the CNS parenchyma and subsequently raised survival rates and minimized neuropathology [160].

Another important cytokine-receptor interaction in the context of CNS infection has been demonstrated between the IL-1 cytokine family and its receptor IL-1R1 [161]. Its involvement during WNV CNS infection has been studied using a mouse model, illustrating an overall more severe infection in mice lacking functional IL-1R1 receptors. The ligand, IL-1*β*, is mainly produced by CD11b^+^CD45^high^ monocytes migrating into the CNS. IL-1*β* functionality is required for correct interaction of CXCR4 and CXCL12, which tightly regulates the migration across an endothelial cell layer. Unaltered interaction between the receptor and its ligand leads to CXCR4^+^ cell retention in the microvascular system of the CNS [113]. Additionally, this cytokine sustains and controls the effector activity of T-lymphocytes but not B-lymphocytes. The functionality of active T-lymphocytes during inflammation has been shown to be reduced only in CD4^+^ but not in CD8^+^ cells. However, CD8^+^ and CD4^+^ T-cells activated by antigen presenting cell (APC) can further be stimulated with IL-1*β*, causing enhanced migration and cytokine synthesis [162, 163]. Interestingly, IL-1*β* was shown to be involved in the inflammasome signaling complex and exhibited increased susceptibility to WNV pathogenesis* in vivo*. Moreover, the reduction of IL-1*β* effects impaired the quality of the effector CD8^+^ T-cell response and reduced antiviral activity within the CNS. IL-1*β* signaling synergized with type 1 IFN to suppress WNV replication in neurons by this controlled virus replication within the CNS [164].

Activated monocytes are also essential for viral clearance and host survival in the CNS. Prior to monocyte migration into the WNV-infected CNS, a rapid infiltration of monocytes into the vascular system can be observed [165]. Furthermore, the importance of CCL2 during monocytosis was underlined, whilst showing that viral clearance is independent of CCL2 binding to CCR2. CD8^+^ T-cells and PMN exhibit a CCL7 dependent migration into the CNS succeeding WNV infection leading to increased viral elimination and survival [165]. However, Ly6C^high^ inflammatory monocytes have shown to cause more severe neuropathological problems once they have migrated to the infected brain during viral CNS disease [166]. The chemokines CCL2 and CCL7, which bind to CCR2 on monocytes, are known to be of substantial importance during inflammatory monocyte release from the bone marrow [167].

In summary, WNV-induced CNS disease involves the release of a broad range of cytokines and chemokines. Particularly, IL-1*β* stands out with its important role in attraction of activated antigen specific immune cells and involvement in regulation of WNV-induced neuroinflammation. Due to a lack of consistent immunological data on WNV infection in animals and humans, further studies are needed to investigate WNV CNS infection. These implications have been critically reviewed by Trobaugh and Green [168]. On a genetic level, significant differences in expression patterns during an acute inflammatory response could be observed between orthologues of mice and human [101]. However, the Collaborative Cross (CC) mouse model has been suggested to mimic the genetic diversity of human appropriately and therefore to be relevant to study genetic variations during WNV infection [102]. Recently, a previously unappreciated phenomenon in WNV infection models has been observed. The authors described notable intragroup variations in the end-point disease in mice infected with different WNV strains of intermediate virulence [100]. Taken together, optimized models need to be identified to verify previous* in vitro* and* in vivo* models to translate observations made in rodents to humans.

### 6.5. Paramyxoviridae

Viruses belonging to the Paramyxoviridae family have a single negative sense stranded RNA genome and include well known species such as mumps (MuV) and measles (MV). In the following, we will highlight primarily findings in measles CNS pathogenesis.

CNS involvement in measles can be classified into primary measles encephalitis, postmeasles encephalitis, measles inclusion body encephalitis, and subacute sclerosing panencephalitis [69]. CXCL10- and CCL5-mediated lymphomonocytic infiltration during measles CNS infection has been demonstrated in a transgenic mouse model [169]. When depleting CXCL10 and/or CCL5, T-cell migration into the brain parenchyma was significantly altered. Up to 40% overall reduction of CD4^+^ and CD8^+^ T-lymphocyte recruitment in mice lacking available CXCL10 and a reduced CD8^+^ but not CD4^+^ T-cell migration after the application of anti-CCL5 antibody was observed. The neuronal presentation and disease outcome of patients is possibly further influenced through the involvement of different cell types in the CNS such as astrocytes and microglia [169].

The second important member of this viral family is the mumps virus. Only scant data exist, demonstrating the role of cytokines, chemokines, and leukocytes in mumps CNS infection. Infected mononuclear cell migration across the BCSFB of newborn hamsters has been shown to mediate meningoencephalitis [70]. Moreover, in clinical studies, children with mumps meningitis exhibit a significantly higher concentration of IL-8, IL-10, IL-12, IL-13, and IFN-*γ* in the CSF compared to children with meningitis caused by other viruses [170].

Interestingly, measles and mumps virus have not enjoyed the same amount of attention with regard to pathogenic research as other neurotropic viruses, despite the high clinical burden. This might be due to the availability of well efficacious vaccines. The cytokine and chemokine profiles and subsequent leukocyte migration should be the focus of future studies.

### 6.6. Togaviridae

This viral family has a linear single-stranded positive sense RNA genome and can naturally be encountered in mosquitos, humans, birds, and mammals. The arthropod-borne CHIKV, belonging to the genus of* Alphaviruses*, has recently been under more thorough investigation, due to several severe epidemics worldwide. The virus has been classified into three genotypes, which evaluate the origin in terms of geographical regions: (1) West Africa, (2) East/Central/South Africa (ECSA), and (3) Asia [171–173]. During outbreaks in 2006, strains isolated from patients showed distinct genome variations, which led to the suggestion that these molecular adaptations (mutations) enhanced the pathogenicity [174]. Usually, neurological problems occur only intermittently; however, with the novel ECSA genotype, higher levels have been observed in neonates as well as in adults. A clinical study elucidated that an array of unusually high neurological complications arose from infection with these CHIKV-strains. In 16.3% of patients, neurological affection was observed. Out of these, 55.1% suffered from encephalitis [175].

The CHIKV has been shown to gain access into the CNS via the olfactory nerve. Intranasal injection of BALB/c mice with the CHIKV causes neuronal degeneration, further leading to tissue necrosis [176]. Scant data exist about the interaction of CHIKV with primary human cells. Studies* in vitro* using endothelial cells hCMEC/D3, epithelial cells A549, lymphocytes, and monocytes indicated very low infection and/or replication rates for CHIKV [177]. Although CHIKV infection of human in* in vitro* models has been analyzed in a few studies, appropriate* in vivo* models for CNS infection are scarce.

Various proinflammatory chemokines, including CCL2, IL-6, and TNF-*α* were shown to be upregulated in the mouse brain after CHIKV infection, enhancing T-cell and macrophage attraction. The authors underline the importance of anti-inflammatory stimulus through IL-4 once the infection has been controlled. IL-4 hinders the breakdown of the BBB which would allow further virus entry and has the additional characteristic of activating T- and B-cells [178]. However, high levels of IL-10, also associated with CHIKV infection in mice, may act as immunosuppressive and impede Th1 and NK cell functionality [179]. The virus has also been shown to target astrocytes in the CNS, both* in vitro* and* in vivo*, which in turn activates dendritic cells [73].

In conclusion, despite some existing data, the understanding of the neuropathogenesis of CHIKV is still very limited. Nevertheless, the research conducted suggests neurotropic characteristics of the virus. However, data on leukocyte activity and immune response following CNS infection with CHIKV hardly exist. Due to an increasing observation of clinical CNS, involvement in this area is of great interest for further research.

## 7. Conclusion and Fields for Future Research

In this review we highlight complex interactions between different CNS-tropic viruses, inflammatory mediators and leukocytes, and the CNS. However, due to the complex nature of controlled biological processes in humans, the task to identify specific cytokine and chemokine profile for each pathogen is demanding. Adequate models and additional* in vitro*,* in vivo*, and clinical studies analyzing CNS inflammation and leukocyte migration in the context of viral CNS infections are warranted. The thorough understanding of the complex and interrelated inflammatory mechanisms as well as identifying universal mediators promoting CNS inflammation is essential for the development of new diagnostic and treatment strategies.

## Figures and Tables

**Table 1:** Classification, characterization, and affected CNS regions of relevant viruses causing infection in the human CNS.

Virus family	Species name	Genome structure	Affected CNS regions	BBB or BCSFB
Retroviridae	Human immunodeficiency virus (HIV) [57]	(+)ssRNA	Meninges, cerebral cortex, white matter, brain stem, ependyma	BBB

Herpesviridae	Varicella zoster virus (VZV) [58]	dsDNA	Meninges, brainstem, subependymal vessels	BBB
	Cytomegalovirus (CMV) [59]	dsDNA	Cerebral cortex, white matter, ependyma, CP	BBB/BCSFB
	Human herpes virus-6 (HH-6) [60]	dsDNA	Front lobe, thalamus, white matter	BBB
	Herpes simplex virus-1 (HSV-1) [61]	dsDNA	Cerebral cortex	BBB
	Epstein Barr virus (EBV) [62]	dsDNA	Meninges, spinal cord, peripheral nerves	BBB

Picornaviridae	Nonpolio enterovirus (NPEV) [63]	(+)ssRNA	CP, cerebellum	BBB/BCSFB
	Poliovirus (PV) [64]	(+)ssRNA	Brain stem	BBB
	Human parechovirus (HPeV) [65]	(+)ssRNA	Meninges, ependyma, CP	BBB/BCSFB

Flaviviridae	Tick-borne encephalitis virus (TBEV) [66]	(+)ssRNA	Cerebral cortex	BBB
	West Nile virus (WNV) [67]	(+)ssRNA	Cerebral cortex, thalamus, hippocampus, brain stem, cerebellum	BBB
	Japanese encephalitis virus (JEV) [68]	(+)ssRNA	Meninges, cerebral cortex	BBB

Paramyxoviridae	Measles virus (MV) [69]	(−)ssRNA	Meninges, cerebral cortex	BBB
	Mumps virus (MuV) [70]	(−)ssRNA	Meninges, ependyma, CP	BBB/BCSFB

Rhabdoviridae	Rabies virus (RV) [71]	(−)ssRNA	Thalamus, hippocampus, brain stem	BBB

Polyomaviridae	John Cunningham virus (JCV) [72]	dsDNA	Subcortical white matter	BBB

Togaviridae	Chikungunya virus (CHIKV) [73]	(+)ssRNA	White matter, CP, meninges	BBB/BCSFB
	Eastern equine encephalitis virus (EEEV) [74]	(+)ssRNA	Brain stem, thalamus	BBB

dsDNA, double-stranded DNA; ssRNA, single-stranded RNA; CP, choroid plexus; BBB, blood-brain barrier; BCSFB, blood-cerebrospinal fluid barrier.
